# The dysfunction in intestinal microorganisms and enzyme activity as significant contributors to diarrhea with kidney-yang deficiency syndrome

**DOI:** 10.3389/fmicb.2023.1324938

**Published:** 2024-01-09

**Authors:** Mengsi Zhou, Xiaoya Li, Xuehong Wang, Na Deng, Ying Cai, Zhoujin Tan

**Affiliations:** ^1^College of Chinese Medicine, Hunan University of Chinese Medicine, Changsha, Hunan, China; ^2^Yunnan Provincial Key Laboratory of Molecular Biology for Sinomedicine, Kunming, Yunnan University of Traditional Chinese Medicine, Kunming, Yunan, China; ^3^The Second Xiangya Hospital of Central South University, Changsha, Hunan, China

**Keywords:** diarrhea with kidney-yang deficiency syndrome, intestinal microorganisms, enzyme activities, oxidative stress, intestinal microbial activity

## Abstract

**Object:**

To investigate the pathogenesis of diarrhea with kidney-yang deficiency syndrome by examining characteristic changes in intestinal microorganisms, enzyme activities, oxidative stress, and metabolism indices.

**Methods:**

Twenty mice were randomly and equally divided into control group (NC) and model group (NM). Mice in NM group received adenine suspension at a dosage of 50 mg/(kg⋅day) by gavage, 0.4 mL/time, once a day for 14 days, and *Folium sennae* decoction at a dosage of 10 g/(kg⋅day) by gavage, 0.4 mL/time, once a day for 7 days, starting on 8th day. Mice in NC group were administered an equivalent amount of sterile water by gavage once a day for 7 days, and twice a day from the 8th day. After modeling, assessments encompassed microbial culture, organ index calculation, microbial and enzyme activity detection, malondialdehyde (MDA) content determination, superoxide dismutase (SOD) activity, blood biochemical tests, and observation of kidney tissue pathological changes.

**Results:**

The results showed that in NM group, a reduction in the number of *Lactobacillus* and *Bifidobacteria* was noted, accompanied by an increase in the number of bacteria and *E. coli*. Xylanase activity in the intestinal contents and mucosa, protease activity in the intestinal mucosa, and intestinal mucosa microbial activity were diminished. Conversely, the activities of amylase, sucrase, and lactase increased in intestinal mucosa. Additionally, there was an elevation in the level of MDA. Renal tubular dilatation and inflammatory cell infiltration were observed in the renal interstitium.

**Conclusion:**

These dysfunctions in intestinal microorganisms and enzyme activities suggest potential involvement in diarrhea with kidney-yang deficiency syndrome.

## 1 Introduction

Diarrhea is a prevalent global health issue. In 2016, the global incidence of diarrhea exceeded 4.4 billion cases, resulting in over 1.6 million deaths and ranking eighth among common causes of mortality. Diarrhea causes huge medical and healthcare costs for patients and has a huge economic impact on society (Wang et al., 2021). The diagnosis of diarrhea is mainly based on abnormal stool morphology, while frequent defecation with normal stool morphology is called pseudo-diarrhea (Schiller et al., 2017). Severe acute diarrhea or chronic diarrhea can have a significant impact on human health through dehydration, malnutrition, a compromised immune system, and socio-economic burden. Mounting evidence suggests that an imbalance in gut microbiota is a significant factor contributing to increased susceptibility to various pathogens and subsequent onset of diarrhea.

The relationship between gut microbiota and diarrhea is complex, involving multiple regulatory mechanisms. Invading pathogens suppress the growth and decimation of beneficial intestinal bacteria, resulting in an imbalance that renders the host more susceptible to a variety of diseases and conditions, including diarrhea. Additionally, certain pathogens produce toxins that disrupt normal intestinal function, triggering an immune response that can lead to diarrhea (Li Y. X. et al., 2021). Several factors contribute to the imbalance of gut microbiota, and one such factor is dietary habits. High-fat and high-protein diets have been observed to impact the composition of intestinal microorganisms. These diets decrease the abundance of beneficial lactic acid bacteria, crucial for maintaining intestinal health. A high-fat diet increases the number, diversity, and richness of Operational Taxonomic Units in mouse intestinal contents, resulting in structural and compositional modifications in the gut microbiota. Fatigue in combination with a high-fat diet disturbs the microbiota, leading to an increase in harmful bacteria and a decrease in beneficial ones. This disruption contributes to elevated inflammatory factors, decreased immune factors, and ultimately the onset of diarrhea. Specifically, the presence of certain bacteria, such as *Corynebacterium*, *Gemella*, and *Methylobacterium*, increases, while beneficial bacteria like *Pediococcus* decrease. *Gemella* is found to be significantly negatively correlated with total cholesterol, highlighting the connection between gut microbiota imbalance, dysregulated lipid metabolism, and diarrhea induced by high-fat diets under fatigue conditions (Li et al., 2022c; Zhou et al., 2022a; Liu et al., 2023; Qiao et al., 2023a). Changes in the microenvironment of intestinal microecological can also contribute to gut microbiota dysbiosis. High temperature and humidity have detrimental effects on the gut microbiota, particularly causing a reduction in the population of *Lactobacillus*, which may be a significant cause of hot and humid diarrhea (Qiao et al., 2023b). Beneficial bacteria play a crucial protective role within the intestine by regulating the composition of the gut microbiota, inhibiting excessive growth of harmful bacteria, and reducing oxidative stress. They achieve this through various mechanisms such as metal ion chelation ability, antioxidant system, regulation of signaling pathways, ROS enzyme production, and modulation of gut microbiota. *Lactobacillus* and *Bifidobacterium*, are well-established probiotics producing lactic acid, acetic acid, and propionic acid, which contribute to maintaining a balanced gut microbiota and suppressing the proliferation of various pathogenic bacteria (Wang et al., 2017). The diversity and richness of the gut microbiota, as well as the interactions between microbes, play crucial roles in regulating intestinal health.

The diversity of gut microbiota correlates with the occurrence of diarrhea. It was observed that the Shannon index, which indicates bacterial diversity, decreased in mice with diarrhea following *Folium sennae* treatment (Zhang et al., 2020). Antibiotic-associated diarrhea (AAD) is also associated with reduced diversity of intestinal mucosal flora (Li et al., 2022a). The diversity of gut microbiota in patients with diarrhea is lower, which may be related to the destruction of the stability of gut microbiota. Metabolites produced by the gut microbiota, such as short-chain fatty acids (SCFAs), play a crucial role in maintaining intestinal health. They contribute to regulating water and electrolyte balance, modulating the equilibrium of the gut microbiota, enhancing intestinal function, exerting anti-inflammatory and anti-tumor effects, and regulating gene expression (Li C. R. et al., 2023). Diarrhea may induce changes in the metabolites of gut microbiota, subsequently affecting intestinal function (Masuda et al., 2023). In the adenine and *Folium sennae*-induced animal model of diarrhea, alterations occurred in the structure and function of the gut microbiota in mice. Signature bacteria, including *Lactobacillus intestinalis* and *Bacteroides acidifaciens*, were enriched, and these were associated with SCFAs, intestinal inflammation, and renal function (Li et al., 2022b). The study found that jujube polysaccharides have a significant ability to restore the dysbiosis of the gut microbiota caused by AOM/DSS, including an increase in the abundance of acetate-producing bacteria (Ji et al., 2020). Ferulic acid from Dendrobium officinale can enhance the expression of tight junction proteins, including ZO-1, Occludin, and Claudin-1, to restore intestinal mucosal barrier function. It can also effectively regulate dysbiosis of the gut microbiota, enhance the production of short-chain fatty acids, contribute to maintaining intestinal homeostasis, inhibit inflammation, and restore intestinal barrier integrity (Wang et al., 2023). With the in-depth exploration of the gut microbiota theory and the advancements of modern technology, adjusting gut microbiota emerges as a strategy for diarrhea treatment. Approaches such as probiotics or fecal microbiota transplantation (FMT) can ameliorate symptoms by increasing beneficial bacteria and restoring the balance of gut microbiota (Wei et al., 2016; Lai et al., 2019; Zhong et al., 2019; Li Q. H. et al., 2023; Masuda et al., 2023).

The dysregulation of intestinal microorganisms and enzyme activity is implicated in the occurrence of diarrhea. Therefore, we hypothesize that the imbalance in intestinal microorganisms and enzyme activity is one of the mechanisms underlying the development of diarrhea with kidney-yang deficiency syndrome. In this experiment, we employed microbiological culture techniques, enzyme activity assays, and microbiota viability measurement techniques to validate this hypothesis. By exploring the changes in gut microbiota and enzyme activity in mice with diarrhea with kidney-yang deficiency syndrome, the aim is to elucidate the underlying mechanisms of diarrhea occurrence from the perspective of gut microbiota and enzyme activity. The results of this study will provide new theoretical insights into understanding diarrhea with kidney-yang deficiency syndrome, offering a scientific basis for clinical treatment.

## 2 Materials and methods

### 2.1 Materials

#### 2.1.1 Animals and feed

Twenty SPF male Kunming mice, weighing 18–22 g, aged 4 weeks, were purchased from Hunan Slix Laboratory Co., LTD., the certificate number of laboratory animal quality: ZS-202106150014. The mice were housed in the Laboratory Animal Center of Hunan University of Chinese Medicine. The experiment was carried out in a barrier environment, with a room temperature of 23–25°C, relative humidity of 50–70%, light/dark cycle for 12 h, and free diet and water. Ordinary mouse feed was provided by Hunan Slaike Jingda Experimental Animal Co., LTD. [No.: SYXK (Xiang)2020-0006]. The experiment complied with the standards of the Animal Ethics and Welfare Committee of the Hunan University of Chinese Medicine (permission number: LLBH-202106120002).

#### 2.1.2 Medicine and preparation

Adenine (Changsha Yaer Biotechnology Co., LTD., batch number: EZ2811A135) was dissolved in sterile water according to the body weight of mice every day and prepared into a suspension of 50 mg/(kg⋅day) of adenine. *Folium sennae* (Anhui Puren Traditional Chinese Medicine Decoction Pieces Co., LTD., batch number: 2005302). We took 28 g *Folium sennae*, soaked it in water for 30 min, decocted it, concentrated the liquid into *Folium sennae* decoction with a crude drug concentration of 1 g/mL, and stored it at 4°C for use.

#### 2.1.3 Reagents and kits

O-nitrobenzene β-D-galactoside pyranoside (ONPG)reagent, 3,5-dinitrosalicylic acid (DNS) reagent, and five substrate solutions were prepared in the lab. Foline-phenol (Hefei Bomei Biotechnology Co., Ltd), Fluorescein diacetate (FDA, Shanghai Yuanye Biotechnology Co., Ltd), Acetone (Hunan Huihong reagent Co., Ltd). MDA kit: Beijing Leagene Biotechnology Co. SOD kit: Beijing Leagene Biotechnology Co.

### 2.2 Methods

#### 2.2.1 Grouping and modeling

After 3 days of adaptive feeding, twenty mice were divided into the NC and NM groups by random number table method, with ten mice in each group. NM group mice were given adenine suspension 50 mg/(kg⋅day) by gavage every morning, 0.4 mL/time, once a day for 14 days. From the 8th day, the mice in NM group were treated with *Folium sennae* decoction 10 g/(kg⋅day) by gavage every afternoon, 0.4 mL/time, once a day for 7 consecutive days. The mice in NC group were given the same amount of sterile water by gavage once a day for 7 consecutive days, and twice daily from the 8th day. The administration time was consistent with that of NM group.

#### 2.2.2 Intestinal contents collection

After all, mice were sacrificed by cervical dislocation, the jejunum to ileum segments were dissected out, and the intestinal contents were scraped with sterile forceps under a sterile environment. The intestinal contents were grouped into 50 mL sterilized centrifuge tubes with glass beads, labeled and weighed, and stored at −20°C for use (Zhou et al., 2022b).

#### 2.2.3 Intestinal mucosa collection

After removal of the intestinal contents from the jejunum to the ileum, the intestinal tract was cut longitudinally with sterile ophthalmic scissors, the residual intestinal contents were washed away in normal saline, and the excess water on the intestinal wall tissue was drained with filter paper. The intestinal mucosa was scraped with a sterile coverslip, grouped into 50 mL sterilized centrifuge tubes with glass beads, labeled and weighed, and stored at −20°C for use (Li X. Y. et al., 2023).

#### 2.2.4 Blood sample collection and detection

Mice in each group were sampled after 12 h of fasting and dehydration. Blood was collected by the eyeball extraction method, and the blood was allowed to stand for 4 h before centrifugation at 3000 rpm for 15 min to separate serum. The levels of uric acid, alanine aminotransferase (ALT), aspartate aminotransferase (AST), and lactate dehydrogenase (LDH) in serum were analyzed by automatic blood biochemistry analyzer (Li et al., 2022c).

#### 2.2.5 Organ collection and organ index calculation

After the mice were weighed, the mice were sacrificed by cervical dislocation on a sterile operating table. The intact spleen, thymus, and liver were removed, and the attached surface fascia and adipose tissue were removed. Spleen, thymus, and liver indexes calculation: organ index = organ weight (mg)/body weight (g) (Li et al., 2022a).

#### 2.2.6 Determination of microbiota in intestinal contents

Bacteria were cultured in beef extract-peptone agar medium. *Lactobacillus* was cultured on deMan Rogosa Sharpe agar medium. *Bifidobacteria* were cultured in a Bifidobacteria agar medium. *E. coli* was cultured on eosin-methylene blue agar medium. Sterile water was added to the centrifuge tube in which the intestinal contents were collected in a sterile environment. The centrifuge tube was placed in a thermostatic oscillator and shaken for 30 min to fully release microorganisms from the intestinal contents. The microorganisms were cultured and counted using the dilution plate culture counting method. Three dilutions were made for each group and each dilution was repeated three times to calculate the number of bacteria per gram of intestinal contents, unit CFU/g. Bacteria and *E. coli* were incubated aerobically at 37°C for 24 h before colony counting. *Lactobacillus* and *Bifidobacteria* were incubated anaerobically for 48 h at 37°C for colony counting (Wu et al., 2021; Qiao et al., 2022).

#### 2.2.7 Determination of intestinal enzyme activity

Under sterile conditions, sterile water was added to the centrifuge tube that collected the intestinal contents and mucosa. The centrifuge tube was shaken in a constant temperature oscillator for 30 min to completely release the enzymes in the intestinal contents and mucosa and then centrifuged at 3000 rpm for 10 min. The DNS colorimetric method was used for amylase, sucrase, and xylanase, and the Folin-phenol method was used for protease, for lactase, the ONPG method was used. Enzyme activities in the supernatant were analyzed with a UV spectrophotometer and calculated per gram of intestinal contents or mucosa in unit U/g (Wu et al., 2021; Qiao et al., 2022).

#### 2.2.8 Determination of intestinal microbial activity

Under sterile conditions, sterile water was added to the centrifuge tube that collected intestinal contents and mucosa in proportion. The centrifuge tube was placed in a constant temperature oscillator and shaken for 30 min to completely release the enzymes in intestinal contents and mucosa, and then centrifuged at 3000 rpm for 10 min. One blank tube and three sample tubes were set up in each group. Blank tube: 2 mL of FDA reaction solution, 2 mL of acetone, and 10 μL of sample were added to a dry sterilized tube, and the mixture was incubated at 24°C for 90 min. Sample tube: 2 mL of FDA reaction solution and 10 μL of sample were added to a dry sterilized tube, the mixture was incubated at 24°C for 90 min before removal, and the reaction was terminated by adding 2 mL of acetone. Finally, the absorbance value was measured with a UV spectrophotometer at a wavelength of 490 nm (Li C. R. et al., 2023).

#### 2.2.9 Determination of MDA and SOD levels in kidney tissues

Malondialdehyde was detected by the thiobarbituric acid microplate method. After the kidney tissue was homogenized and lysed with phosphate buffer, the mice were centrifuged at 1600 rpm for 10 min at 4°C, and the supernatant was removed and placed on ice until measured. MDA content in kidneys was measured strictly according to the manufacturer’s instructions, the absorbance at 535 nm was measured using a microplate reader, and MDA concentration was calculated.

Superoxide dismutase was detected by the nitro-blue tetrazolium riboflavin microplate method. According to the ratio of 500 μL SOD extract per 100 mg tissue, the tissue was homogenized by a tissue homogenizer at 4°C, centrifuged at 4000 rpm for 10 min, and the supernatant was measured strictly according to the manufacturer’s instructions. At the end of the reaction, the absorbance at 560 nm was measured by a microplate reader, and the SOD activity was calculated.

#### 2.2.10 Histological observation of kidneys

The mice were sacrificed by cervical dislocation and dissected immediately. The kidney tissues were removed and fixed immediately in 4% paraformaldehyde solution and stored at room temperature or 4°C. The fixed tissues were dehydrated and transparent in ethanol and xylene, then embedded in paraffin, sectioned, and stained with hematoxylin and eosin. The histopathological changes in the kidney were observed under a light microscope (Zhu et al., 2022).

#### 2.2.11 Statistical analysis

SPSS 25.0 software was employed for data processing and statistical analysis. The measurement data for each group were presented as mean ± standard deviation (mean ± SD) following a normal distribution. Normality tests were applied for samples with normal distribution, while the Mann-Whitney *U*-test was utilized for non-normally distributed data. In cases of comparing more than two groups, a one-way analysis of variance was conducted for samples with normal distribution and homogeneity of variance. The LSD method was employed for subsequent comparisons, and the Kruskal-Wallis test was used for non-normally distributed or heterogeneous variance samples. Categorical data were presented as percentages (%), and intergroup comparisons were performed using the chi-square test. The significance level (α) was set at 0.05. A *P*-value less than 0.05 was considered statistically significant.

## 3 Results

### 3.1 Effects of diarrhea with kidney-yang deficiency syndrome on organ indexes in mice

**FIGURE 1:**
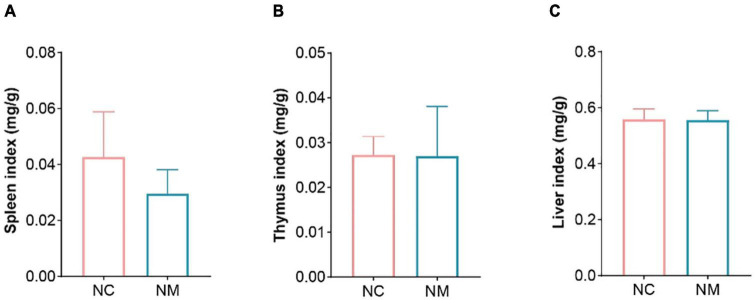
Effects of diarrhea with kidney-yang deficiency syndrome on organ index in mice. **(A)** Spleen index. **(B)** Thymus index. **(C)** Liver index. (NC: control group, NM: model group).

The indexes of the spleen, thymus, and liver can reflect the strength of immune function to a certain extent. According to Figure 1, there was no significant difference in the indexes of the spleen, thymus, and liver between the NM and NC groups (*P* > 0.05), and the spleen index of NM group showed a downward trend.

### 3.2 Effects of diarrhea with kidney-yang deficiency syndrome on blood biochemical indexes in mice

**FIGURE 2:**
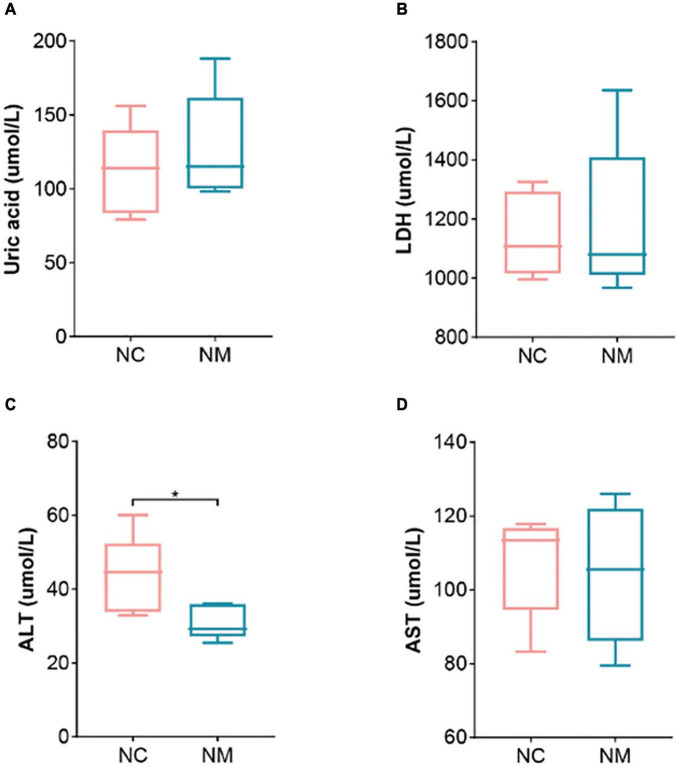
Effect of diarrhea with kidney-yang deficiency syndrome on blood biochemical indexes in mice. **(A)** Uric acid. **(B)** Lactate dehydrogenase. **(C)** Alanine aminotransferase (ALT). **(D)** Aspartate aminotransferase (AST) (NC: control group, NM: model group, *P < 0.05).

Aminotransferase and AST are important indicators for evaluating liver function. Figure 2 shows that ALT in NM group was lower than that in NC group (*P* < 0.05), and there was no significant difference in AST (*P* > 0.05). Uric acid is the end product of purine metabolism, which can reflect the metabolic function of the kidney to a certain extent. In this experiment, there was no significant difference between the two groups (*P* > 0.05). LDH is a cytoplasmic enzyme widely expressed in tissues, and its activity level can reflect the tissue’s oxygen supply and energy metabolism status. An elevated level of LDH in the serum is an important indicator of tissue and cell damage. The relationship between yang deficiency and energy metabolism is closely related. A decrease in LDH indicates abnormal energy metabolism, with inhibited glucose oxidation for energy supply (Xue et al., 2001). Therefore, animals in a yang deficiency state may exhibit symptoms such as decreased body temperature and aversion to cold. In this experiment, there was no significant difference between the two groups (*P* > 0.05).

### 3.3 Effects of diarrhea with kidney-yang deficiency syndrome on kidney tissue in mice

**FIGURE 3:**
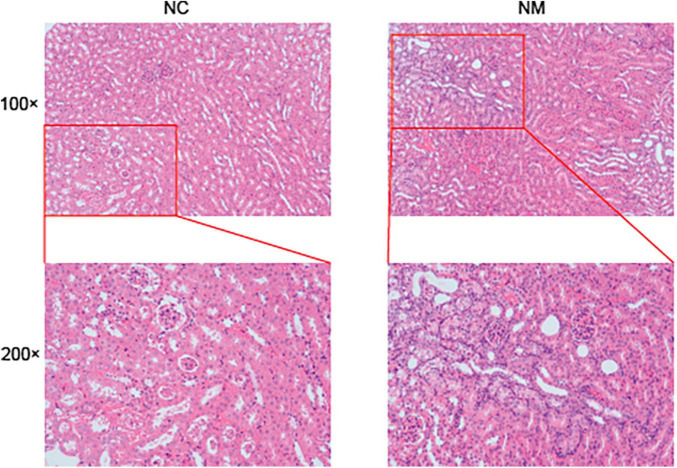
Effect of diarrhea with kidney-yang deficiency syndrome on kidney tissue in mice (NC: control group, NM: model group, 100 × :100 times magnification, 200 × :200 times magnification).

According to Figure 3 of the kidney tissue section, the morphology and structure of nephrons in NC group were normal, and no pathological changes were observed. The kidney tissue of mice in NM group had changes such as renal tubular dilatation and renal interstitial inflammatory cell infiltration.

### 3.4 Effects of diarrhea with kidney-yang deficiency syndrome on MDA and SOD in the kidney of mice

**FIGURE 4:**
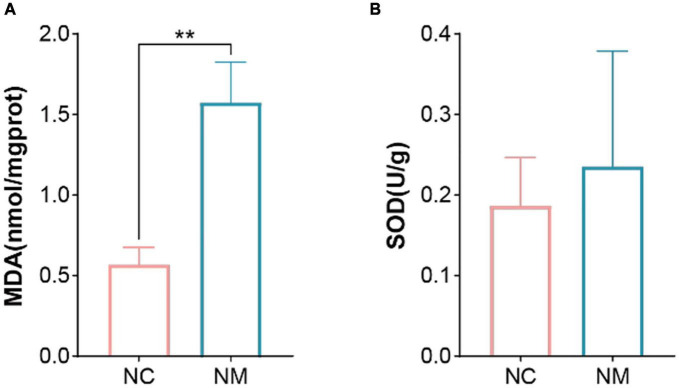
Effects of diarrhea with kidney-yang deficiency syndrome on MDA and SOD in the Kidney of Mice. **(A)** Malondialdehyde (MDA). **(B)** Superoxide dismutase (SOD). (NC: control group, NM: model group, **P < 0.01).

Malondialdehyde is a natural product of lipid oxidation in organisms. Some fats are gradually decomposed into a series of complex compounds including MDA after acidification and oxidation. The level of MDA can be measured to detect the level of lipid oxidation, so it is often used as an indicator of lipid oxidation. SOD is an important antioxidant enzyme in the organism, which can catalyze the dismutation of superoxide anion to produce hydrogen peroxide and oxygen. Figure 4 shows that compared with NC group, the MDA content of NM group was significantly increased (*P* < 0.05), and the SOD activity only showed an upward trend (*P* > 0.05).

### 3.5 Effects of diarrhea with kidney-yang deficiency syndrome on intestinal microorganisms in mice

**FIGURE 5:**
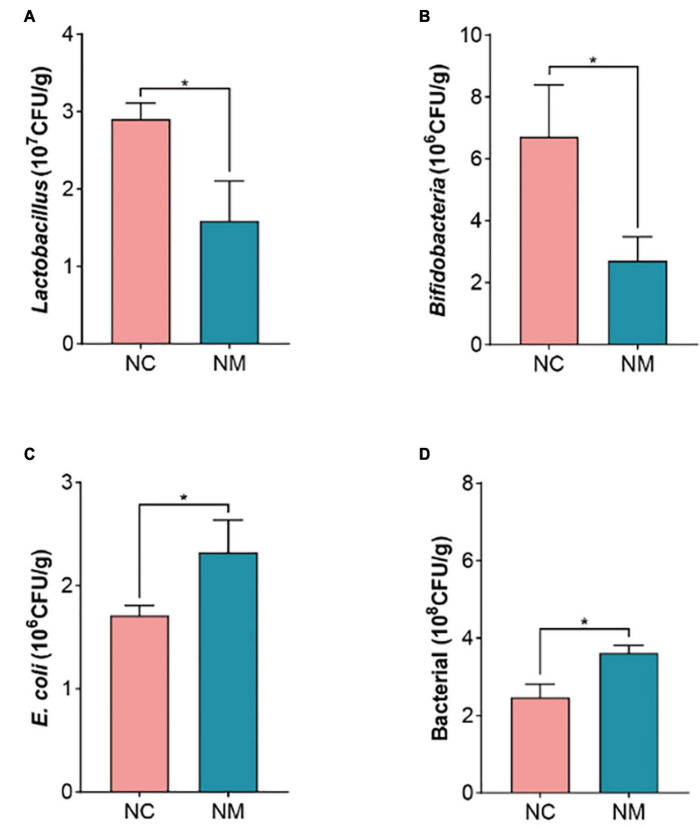
Effects of diarrhea with kidney-yang deficiency syndrome on gut microbiota in mice. **(A)** Lactobacillus. **(B)** Bifidobacteria. **(C)** E. coli. **(D)** BacteriaL. (NC: control group, NM: model group, *P < 0.05).

The occurrence of diarrhea is usually associated with a decrease in beneficial bacteria and an increase in harmful bacteria. The results showed that the number of *Lactobacillus* and *Bifidobacteria* decreased (*P* < 0.05) and the number of bacteria and *E. coli* increased (*P* < 0.05) in NM group, as shown in Figure 5. It is suggested that diarrhea with kidney-yang deficiency syndrome affects the gut microbiota of mice, resulting in the reduction of the number of beneficial bacteria and the increase of harmful bacteria, and the original gut microbiota balance is broken.

### 3.6 Effects of diarrhea with kidney-yang deficiency syndrome on intestinal enzyme activities in mice

**FIGURE 6:**
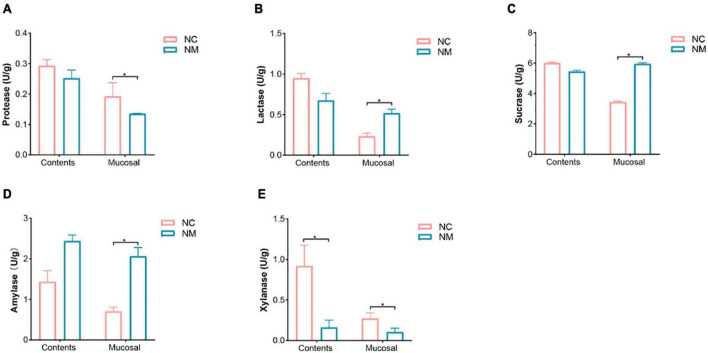
Effects of diarrhea with kidney-yang deficiency syndrome on intestinal enzyme activities in mice. **(A)** Protease. **(B)** Lactase. **(C)** Sucrase. **(D)** Amylase. **(E)** Xylanase. (NC: control group, NM: model group, *P < 0.05).

The activity of intestinal enzymes is closely related to the digestion and absorption function of the intestine. Lactase, sucrase, and xylanase can be produced by intestinal microorganisms, and their activity levels reflect the state of these microorganisms. In this experiment, depicted in Figure 6, the activities of protease, amylase, lactase, and sucrase in intestinal contents showed no significant differences between the two groups (*P* > 0.05). In contrast, amylase, lactase, and sucrase activities in intestinal mucosa were notably higher in NM group compared to NC group (*P* < 0.05), while the protease activity was significantly lower in NM group than in NC group (*P* < 0.05). Xylanase activity in both intestinal contents and mucosa was significantly lower in NM group compared to NC group (*P* < 0.05). The results suggest that the activities of protease, amylase, lactase, and sucrase in intestinal contents of mice with diarrhea with kidney-yang deficiency syndrome do not change significantly, but these enzyme activities notably differ in the intestinal mucosa. Alterations in xylanase activity were observed in both intestinal contents and intestinal mucosa.

### 3.7 Effect of diarrhea with kidney-yang deficiency syndrome on intestinal microbial activity in mice

**FIGURE 7:**
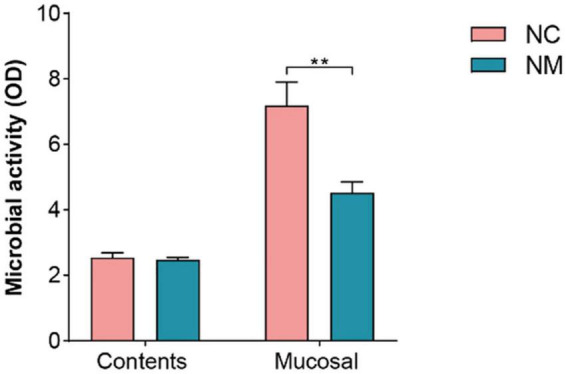
Effect of diarrhea with kidney-yang deficiency syndrome on intestinal microbial activity in mice (NC: control group, Mucosal: NM: model group, **P < 0.01).

The FDA hydrolysis method can be used to detect the activity of microorganisms in the intestinal tract, which can reflect the overall metabolism of intestinal microorganisms. In this experiment, there was no significant difference in microbial activity in the intestinal contents between the two groups of mice (*P* > 0.05), but the microbial activity in the intestinal mucosa was lower in NM group than in NC group (*P* < 0.05), as shown in Figure 7. These results indicated that there was no significant change in the overall microbial metabolism level in the intestinal contents of mice with diarrhea with kidney-yang deficiency syndrome, but the overall microbial metabolism level in the intestinal mucosa of NM group mice was decreased.

## 4 Discussion

### 4.1 Effects of diarrhea with kidney-yang deficiency syndrome on intestinal microorganisms and enzyme activities in mice

The results revealed that diarrhea with kidney-yang deficiency syndrome had a notable impact on the composition and enzyme activities of intestinal microorganisms in mice. Specifically, a reduction in the abundance of two beneficial bacteria, *Lactobacillus* and *Bifidobacteria*, was observed in the intestinal contents of mice with diarrhea caused by kidney-yang deficiency. Concurrently, there was an increase in the quantity of *E. coli*, recognized as harmful bacteria. *Lactobacillus* and *Bifidobacteria*, both belonging to the lactic acid bacteria genera, are closely related to human health. They represent the most prevalent probiotics, demonstrating potential therapeutic efficacy in various gastrointestinal disorders, including inflammatory bowel disease (IBD) (especially intestinal bursitis), AAD, *Clostridium difficile* toxin-induced colitis, infectious diarrhea, irritable bowel syndrome (IBS), and allergy (Elzouki, 2016). Research indicates a significant reduction in *Bifidobacterium* levels in the feces of IBS patients. Nevertheless, this decrease is considered a consequence rather than a cause of IBS (Kerckhoffs et al., 2009). Dietary factors can adversely influence gut microbiota composition. Notably, a lard-based diet was found to impede the growth of *Lactobacillus* and *Bifidobacterium*. This altered gut microbiota abundance may contribute to an elevated risk of obesity, non-alcoholic fatty liver disease, and atherosclerosis. The depletion of probiotics disrupts intestinal homeostasis and mucosal barrier function, and promotes endotoxin production and growth, thereby leading to intestinal inflammation (Qiao et al., 2022). *E. coli* is a Gram-negative bacterium commonly found in the intestines of humans and animals and can produce toxins during infection, causing symptoms such as diarrhea, fever, urinary tract infections, pain, and bowel cancer. It also plays a crucial role in the occurrence and development of IBD (Khan et al., 2019). The found uncovered that *Folium sennae*-induced diarrhea was associated with the remodeling of intestinal bacteria characteristics and metabolic abnormalities. Notably, there was a significant increase in the abundance of certain bacterial communities, including *Paraprevotella*, *Streptococcus*, *Epulopiscium*, *Sutterella*, and *Mycoplasma*, while others such as *Adlercreutzia*, *Lactobacillus*, *Dehalobacterium*, *Dorea*, and *Oscillospira* were significantly reduced in abundance (Zhang et al., 2020).

Probiotic supplementation with different strains of *Lactobacillus* and *Bifidobacterium* was beneficial in treating infectious diarrhea in young children caused by rotavirus (Boronat et al., 2020). In the case of IBS, *Bifidobacterium Breve* in combination with *Lactobacillus Plantarum* reduces pain and symptom severity in patients with IBS (Saggioro, 2004). Additionally, prebiotics, oligofructose, and inulin can selectively stimulate *Bifidobacterium*, and increase bowel movement frequency, which has a positive effect on IBS with constipation (Gibson et al., 1995). It is important to note that chronic alcohol consumption can alter the composition of gut microbiota. Alcohol promotes the growth of Gram-negative bacteria, reduces *Bacteroidetes* and *Firmicutes*, and increases *Actinobacteria* and *Proteobacteria*. Probiotic use, on the other hand, promotes the growth of anaerobes and Gram-positive bacteria while limiting the growth of Gram-negative bacteria. This helps prevent pathogen attachment and reduces liver damage caused by the production of endotoxins and other toxic compounds by bacteria (Malaguarnera et al., 2014). A review on the therapeutic use of probiotics, specifically Bifidobacterium, highlighted various health benefits associated with their consumption. These benefits include anti-infective activity, antiviral activity, anti-tumor activity, anti-inflammation, promotion of mental health, reduction of fat accumulation, improved nutrient absorption, promotion of bone health, and regulation of the host immune system (Chen et al., 2021).

In the present study, the effects of diarrhea with kidney-yang deficiency syndrome on the gut microbiota were found to be consistent with previous findings in diarrhea cases. This includes an increase in the number of harmful bacteria *E. coli* and a decrease in the number of beneficial bacteria such as *Lactobacillus* and *Bifidobacterium*. It is worth noting that various factors, such as individual differences, diet, and environment, can also influence the composition of gut microbiota. Therefore, further research is necessary to better understand the complex interactions between gut microbiota and diarrhea with kidney-yang deficiency syndrome, as well as the potential therapeutic significance of modulating microbiota for this disease.

Furthermore, in mice with diarrhea with kidney-yang deficiency syndrome, the activity of microbial in intestinal mucosa was found to be decreased. Microbial activity often represents the overall activity of microbial hydrolases and serves as an important indicator of the decomposition ability of microbial matter. it is also a typical indicator used to study the characteristics of microbial communities in natural samples (Li X. Y. et al., 2021). Our group has successfully applied FDA hydrolase activity to assess the overall activity of gut microbiota in animals and humans, although this method was initially developed for soil microbial activity evaluation (Li C. R. et al., 2023). FDA is hydrolyzed by non-specific enzymes (esterases, proteases, lipases, etc.) in bacteria and fungi, and its hydrolysis activity is directly proportional to the number of microbial populations (Yi et al., 2023). Intestinal mucosal microbial activity was found to be decreased in mice with diarrhea induced by a high-fat and high-protein diet (Zhou et al., 2023). However, chronic exposure to high doses of cadmium was found to increase the microbial activity of the intestinal mucosa (Li X. Y. et al., 2021). This experiment observed a reduction in microorganism activity within NM group’s intestinal mucosa. This suggests that diarrhea with kidney-yang deficiency syndrome affected the material decomposition ability of intestinal microorganisms in mice. Additionally, it indirectly signifies a decrease in the overall decomposition ability of microorganisms, specifically esterase, protease, and lipase, in the intestinal mucosa.

Subsequently, alterations in enzyme activities were observed in the intestinal contents and mucosa of mice experiencing diarrhea with kidney-yang deficiency syndrome. Intestinal microorganisms actively participate in lactase and sucrase synthesis. Imbalances in the gut microbiota during diarrhea result in reduced production of lactase and sucrase-producing bacteria, consequently diminishing the activity of these enzymes (Li X. Y. et al., 2023). *Bacillus*, a prominent intestinal microorganism, synthesizes proteases and xylanases and serves as a precursor for cellulase produced by *Bacteroides*, *Clostridium*, and *prebacteroides*. Sustained consumption of vegetable oil or lard leads to the accumulation of acidic metabolites generated by intestinal microorganisms. This accumulation lowers the intestinal pH, subsequently decreasing the activity of proteases, xylanases, amylases, and cellulases (Qiao et al., 2022). Notably, cellulases and xylanases are excreted by intestinal microorganisms, and high doses of aflatoxin B_1_ can significantly augment the activity of xylanases and cellulases (He et al., 2018a). The well-known formula Qiwei Baizhu San improved diarrhea by increasing lactase activity, primarily by enhancing the abundance of lactase-producing bacteria with key lactase genes (He et al., 2018b). The activity of digestive enzymes can partially indicate alterations in intestinal microorganisms. The xylanase activity in intestinal contents of mice with diarrhea with kidney-yang deficiency syndrome decreased. Conversely, the activities of amylase, sucrase, and lactase in intestinal mucosa increased, while protease activity decreased. These results indicate alterations in the digestive and absorption function of the intestine in mice with diarrhea with kidney-yang deficiency syndrome. These changes result from alterations in the gut microbiota of mice with diarrhea with kidney-yang deficiency syndrome. Furthermore, the intestinal contents and mucosa flora exhibit differences, indicating varied changes in isoenzyme activity across different regions.

In summary, diarrhea, intestinal microorganisms, and enzyme activities exhibit interrelated dynamics. Various diarrhea syndromes induce distinct effects on the structure and composition of gut microbiota. The characteristic bacteria, abundance, and diversity of gut microbiota, as measured by high-throughput sequencing technology vary. Additionally, the affected enzymes exhibit different emphases. These findings lay the experimental foundation for the theory that “different bacteria treat different bacteria.”

### 4.2 Effects of diarrhea with kidney-yang deficiency syndrome on the functions of various organs in mice

The organ index denotes the organs’ weight as a percentage of body weight and serves as an indicator reflecting organ development and health to some extent. Following an injury to the animal body, changes in internal organ mass occur, impacting the organ index. The spleen and thymus, vital immune organs, play crucial roles spleen serves as the site for immune cell settlement and response, while the thymus is the primary site for T cell development. Increased body mass is observed in hypersensitivity reactions. Consequently, the spleen and thymus indexes can be fundamental metrics for assessing drug-inhibitory effects on immune organs. An enlarged organ index may signify hyperemia, edema, hyperplasia, hypertrophy, and other lesions. Conversely, a diminished organ index suggests organ atrophy or other damage. In this experiment, although the spleen, thymus, and liver indexes of mice in NM group did not exhibit significant change, the spleen index displayed a declining trend. This suggests that diarrhea with kidney-yang deficiency syndrome inflicted specific damage on the spleen of mice. According to Traditional Chinese Medicine (TCM), diarrhea is associated with spleen function, and kidney-yang deficiency syndrome mostly occurs in the predawn, also known as “ predawn diarrhea,” often occurs during the predawn hours due to the fire of the gate of life deficiency, the fire is not warm, the spleen’s transportation and transformation dysfunction and the inability of the intestinal tract to retain and absorb water. The syndrome of spleen deficiency in TCM was just presented by the decrease of spleen index in this experiment, bridging the perspectives of traditional and modern medicine. Merely attributing spleen deficiency syndrome to a decline in the immune organ index is overly simplistic. Therefore, researchers, through a literature review, have made progress in confirming the quality of spleen deficiency from diverse perspectives using modern medical methods. This paper meticulously elucidates spleen deficiency syndrome as a comprehensive manifestation of reduced functions of the immune system, endocrine system, nervous system, blood system, water and salt metabolism, energy conversion, and digestive system. Simultaneously, it points out that there are too many biological indexes of spleen deficiency syndrome studied by modern medicine, without specificity and pertinence, and correlation between the indexes (He et al., 2015).

The intestinal microbiota plays a crucial role in the occurrence of diarrhea, and it is particularly sensitive to oxidative stress (Guo et al., 2020). When the balance between pro-oxidation and antioxidation is disrupted, the excessive generation of free radicals leading to lipid peroxidation can cause damage to biological membranes. This process is closely associated with the pathological reasons for deficiency in kidney-yang. Oxidative stress may contribute to the development of diarrhea with kidney-yang deficiency syndrome by inducing dysbiosis in the intestinal microbiota and damaging the intestinal epithelial barrier (Wang et al., 2016). Oxidative stress arises when the concentration of reactive oxygen species surpasses the body’s inherent antioxidant defense (Burton and Jauniaux, 2011). MDA is the end product of lipid peroxidation. It serves as a versatile biomarker for assessing oxidative stress in diverse bodily fluids, including blood, urine, and exhaled condensates, across various diseases such as cancer, cardiovascular, pulmonary, and neurodegenerative diseases (Pérez-Hernández et al., 2017; Cordiano et al., 2023). SOD is the sole enzyme with the capability to directly neutralize free radicals, functions as the primary defense against oxidative stress, and is integral to various biological processes (Balamurugan et al., 2018; Borgstahl and Oberley-Deegan, 2018). Both SOD and MDA are widely employed indicators of physiological stress and cellular oxidative damage (Liu et al., 2023). Elevated biomarkers of oxidative damage do not consistently signify heightened oxidative stress; they may also result from malfunctioning repair or replacement systems (Halliwell, 2007). Research indicates that the Tianhuang formula can enhance *Lactobacillus* abundance, along with its metabolite 5-methoxyindole acetic acid, thereby reducing oxidative stress and ameliorating Non-alcoholic fatty liver disease (NAFLD) through the regulation of gut microbiota (Luo et al., 2022). *L. paracasei* Jlus66 (Jlus66), a probiotic isolated from “milk bumps,” enhances the abundance of Gram-positive bacteria like Firmicutes in the gut microbiota. It diminishes Gram-negative bacteria such as Bacteroidetes, Proteobacteria, and Fusobacteria, lowers endotoxin concentration in serum, enhances the structure of gut microbiota, and boosts SOD activity in serum by 29.1%. The protective efficacy of Jlus66 against high-fat diet-induced oxidative damage in rats is affirmed by reductions in serum and liver MDA concentrations, thus mitigating oxidative stress and inflammation and ameliorating NAFLD (Wang et al., 2019). We assessed oxidative stress levels by quantifying MDA level and SOD activity. In this experiment, the MDA level in kidney tissue of NM group was significantly elevated compared to NC group, while the SOD activity exhibited an upward trend. This indicates an increased level of oxidative stress in the kidney of mice with diarrhea with kidney-yang deficiency syndrome, potentially indicating a malfunction in the kidney’s repair or replacement system.

Lactate dehydrogenase is a cytoplasmic enzyme widely expressed in tissues, and its activity level can reflect the tissue’s oxygen supply and energy metabolism status. An elevated level of LDH in the serum is an important indicator of tissue and cell damage. The relationship between yang deficiency and energy metabolism is closely related. A decrease in LDH indicates abnormal energy metabolism, with inhibited glucose oxidation for energy supply. Therefore, animals in a yang deficiency state may exhibit symptoms such as decreased body temperature and aversion to cold. In addition to the symptoms of diarrhea, the animal model used in this experiment also exhibited decreased body temperature and huddling behavior. Therefore, in this experiment, LDH is used to reflect the energy metabolism status of mice with diarrhea with kidney-yang deficiency syndrome.

Adenine is commonly employed in establishing animal models for chronic kidney disease and kidney-yang deficiency syndrome (Khan et al., 2022; Li et al., 2022b). In this study, we investigated the impact of diarrhea with kidney-yang deficiency syndrome on the murine kidneys by assessing serum uric acid levels. The majority of uric acid is endogenously produced, primarily originating in the liver and subsequently in the small intestine. Endogenous uric acid production is influenced by purine diet uptake, *de novo* biosynthesis of purine bases, and degradation and recycling of corresponding nucleotides. The kidney plays a pivotal role in regulating circulating uric acid levels, reabsorbing approximately 90% of filtered uric acid. Urate reabsorption is generally considered a tertiary active transport process, contingent on sodium reabsorption. Moreover, the kidneys are responsible for 60–70% of total uric acid excretion (Bobulescu and Moe, 2012). Uric acid homeostasis refers to the state of relatively stable uric acid levels in the body, determined by the delicate equilibrium between uric acid production and excretion. Excessive uric acid production or inadequate excretion leads to elevated circulating levels, resulting in hyperuricemia. Beyond saturation limits, leads to the deposition of uric acid in the blood, joints, tissues, and urine. Conversely, low serum uric acid causes hypouricemia, prompting the deposition of xanthine and hypoxanthine crystals in joints, muscles, and/or kidneys. Both hyperuricemia and hypouricemia are associated with various chronic diseases, including chronic kidney disease (Dissanayake et al., 2020). Experimental studies have revealed a U-shaped relationship between uric acid levels and measured glomerular filtration rate, demonstrating that lower uric acid levels are not necessarily better for renal function (Jung et al., 2020). In the examined model, no significant change in serum uric acid was observed in NM group compared with NC group. This may be attributed to the unaffected renal filtration and secretion function of uric acid in mice with diarrhea with kidney-yang deficiency syndrome, possibly due to the modeling drugs or a balanced state of uric acid secretion and excretion in the model mice.

When liver cells are damaged or destroyed, liver enzymes such as ALT, present in liver cells are released into the blood, resulting in an elevated ALT level in the blood. Therefore, the measurement of ALT levels in the blood is often utilized to assess liver health and functional status—severe necrosis and destruction of the liver further result in an increased serum concentration of AST. In a study investigating the protective effect of protocatechuic acid on metabolic-associated fatty liver disease, it was demonstrated that catechuic acid could reduce the relative abundance of *Enterococcus*. Correlation analysis indicated a positive correlation between *Enterococcus* and serum liver injury indicators ALT and AST. Overgrowth of *Enterococcus* faecalis exacerbated alcoholic liver disease in mice, promoting inflammation. To comprehend the role of *Enterococcus* faecalis in fatty liver development, investigators conducted FMT experiments, revealing that *Enterococcus* faecalis caused liver inflammation, fat deposition, insulin resistance, as well as decreased carnitine palmitoyltransferase-1α expression (Tan et al., 2023). On the one hand, the gut microbiota plays a crucial role in influencing the occurrence and progression of metabolic liver diseases. Conversely, it impacts hepatocyte regeneration by intricately modulating key cytokines, including interleukin-6, tumor necrosis factor-α, and hepatocyte growth factor, among others, and the levels of essential metabolites in the liver’s metabolic processes, such as bile acids, lipopolysaccharide, and short-chain fatty acids (Xu et al., 2022). In this experiment, we measured serum ALT and AST levels to scrutinize the impact of modeling drugs on the liver. The findings revealed that the serum ALT concentration in NM group was lower than that in NC group, while AST exhibited no significant change between the two groups. This suggests that the modeling drugs did not cause significant damage to the liver, the pivotal detoxification organ. Notably, alterations in gut microbiota in mice with diarrhea with kidney-yang deficiency syndrome demonstrated minimal impact on the liver through the gut-liver axis.

In this experiment, the effect of diarrhea with kidney-yang deficiency syndrome in mice primarily manifested in alterations to intestinal microorganisms and enzyme activities, exhibiting limited immune function, oxidative stress, and the functionality of the liver and kidney. Future investigations should delve into the mechanism through which kidney-yang deficiency syndrome affects intestinal microorganisms and enzyme activities in mice. Additionally, a thorough exploration of immune function and oxidative stress level of kidney-yang deficiency syndrome is warranted to unravel potential pathogenic pathways leading to diarrhea associated with kidney-yang deficiency syndrome. These endeavors aim to lay the necessary experimental groundwork for developing effective treatments. Further experiments could potentially involve comparing the study of intestinal microbiota with reference databases of healthy intestinal microbiota, such as the mouse gut microbiobank (mGMB). This would allow for a more in-depth exploration of information regarding microbial community changes.

## 5 Conclusion

In this investigation, we utilized microbial culture, enzyme activity detection, and microbial activity detection technology to substantiate alterations in the intestinal microorganisms and enzyme activities among mice with diarrhea with kidney-yang deficiency syndrome. The findings indicate a reduction in beneficial bacteria, an elevation in harmful bacteria, and a disruption of the intestinal microecological balance. Consequently, there is a discernible decrease in xylanase activity in intestinal contents and an escalation in amylase, sucrase, and lactase activities in intestinal mucosa. Concurrently, protease activity witnessed a decline, and overall microorganism activity diminished. The observed shifts underscore the pivotal role of intestinal microbial dynamics in the progression of diarrhea with kidney-yang deficiency syndrome, offering a foundational framework for potential interventions such as probiotics or fecal microbiota transplantation. This, in turn, facilitates a deeper comprehension of the mechanisms governing the onset and evolution of diarrhea, thereby guiding clinical approaches.

## Data availability statement

The original contributions presented in the study are included in the article/supplementary material, further inquiries can be directed to the corresponding authors.

## Ethics statement

The animal study was approved by the LLBH-202106120002/Animal Ethics and Welfare Committee of Hunan University of Chinese Medicine. The study was conducted in accordance with the local legislation and institutional requirements.

## Author contributions

MZ: Writing – original draft. XL: Writing – review and editing. ND: Writing – review and editing. XW: Writing – review and editing, Funding acquisition. YC: Funding acquisition, Writing – review and editing. ZT: Writing – review and editing, Supervision.
